# A Rare Case of a Petersen’s Hernia During Pregnancy After Roux-en-Y Gastric Bypass

**DOI:** 10.7759/cureus.80131

**Published:** 2025-03-06

**Authors:** Shalini J Weerasekera, Hasthika Ellepola

**Affiliations:** 1 Obstetrics and Gynecology, Logan Hospital, Logan, AUS

**Keywords:** abdominal pain in pregnancy, bariatric surgery and pregnancy, bariatric surgery and small bowel obstruction, emergency caesarean section, explorative laparotomy, laparoscopic roux-en-y gastric bypass, nausea and vomiting in pregnancy, pregnancy, preterm delivery, roux-en-y complication

## Abstract

Obesity is an escalating problem worldwide and is associated with increased adverse maternal and perinatal outcomes in pregnancy. Bariatric surgery offers women an effective method of sustained weight loss, with patients often less likely to develop obesity-related comorbidities in pregnancy, including hypertension and gestational diabetes. However, despite beneficial effects, previous bariatric surgery can also predispose pregnant patients to developing internal hernias. We present a rare case of a Petersen’s hernia diagnosed in a patient with a previous Roux-en-Y gastric bypass procedure during her third trimester.

## Introduction

Obesity is a growing worldwide epidemic, with significant short-term and long-term adverse outcomes in pregnancy. Obese patients are at significantly higher risk of developing various perinatal complications, including gestational diabetes, pre-eclampsia, preterm birth, fetal macrosomia, and perinatal death [1]. Bariatric surgery offers women a very effective method of significant weight loss with improved fertility health benefits and a reduced likelihood of obesity-related complications in pregnancy. Roux-en-Y gastric bypass is often considered the gold standard bariatric surgery due to superior clinical outcomes and shorter recovery times. However, despite the benefits of procedures like the Roux-en-Y gastric bypass, life-threatening complications can occur in pregnancy, such as internal herniation and potential clinical sequelae [2].

The increased risk of internal hernia formation after bariatric surgery in pregnancy is postulated to be due to the increase in intra-abdominal pressure and displacement of the small bowel with the expanding uterus [2,3]. One study reported an incidence of small bowel obstruction secondary to internal herniation in pregnant patients post Roux-en-Y gastric bypass surgery as 2-11% [4]. Internal hernias arise when weight loss causes a rapid reduction in intra-peritoneal fat, thereby enlarging surgically created mesenteric defects and causing the loosening of mesenteric sutures post bariatric surgery [5,6]. Following Roux-en-Y gastric bypass surgery, the number of potential mesenteric defects differs based on whether an antecolic or retrocolic approach is adopted. This refers to whether the Roux limb is passed either anterior (antecolic) or posterior (retrocolic) to the transverse colon to reach the gastroesophageal junction. In antecolic and retrocolic approaches, the bowel can migrate and can commonly herniate through mesenteric defects in Petersen’s space and intermesenteric jejuno-jejunostomy defects [4]. In the retrocolic approach, herniation can additionally occur through transverse mesocolon defects [4]. This can lead to life-threatening consequences, including intestinal obstruction, volvulus formation, perforation, bowel necrosis, and sepsis [7]. In pregnant patients, internal herniation post-bariatric surgery is associated with significant morbidity and mortality for both mother and fetus.

Pregnant patients with previous bariatric surgery presenting with internal herniation most commonly present with epigastric pain, nausea, and vomiting [8]. However, these symptoms are not uncommon in pregnancy, and thus internal herniation is often a missed or delayed diagnosis in this patient cohort. This can result in lethal maternal-fetal outcomes, and thus, a high index of suspicion for internal herniation must be maintained for pregnant patients with a history of bariatric surgery presenting with abdominal pain.

Very few case reports exist that describe internal hernias in pregnant patients post Roux-en-Y gastric bypass. We present a rare case of a Petersen’s hernia diagnosed in a patient with a previous Roux-en-Y gastric bypass procedure during her third trimester, warranting concurrent preterm delivery and surgical intervention.

## Case presentation

A 40-year-old female G5P3 at 32 weeks and five days gestation presented to the maternity assessment unit at a rural hospital with epigastric pain, nausea, vomiting, and not having passed flatus in four days. She reported normal fetal movements and had no vaginal bleeding or fluid loss. She had previously presented at 28 weeks and at 32 weeks and two days of gestation with epigastric pain, which was presumed to be secondary to gastritis. She had previously had a vaginal birth, followed by two lower segment Caesarean sections. She had previously had a laparoscopic Roux-en-Y gastric bypass procedure two years prior for weight loss and had well-controlled seasonal asthma. She was taking a protein pump inhibitor but otherwise not taking any medication. She had no history of smoking, alcohol use, or illicit drug use. On examination, her abdomen was distended with epigastric tenderness elicited on palpation; however, there was no evidence of peritonism. There were no bowel sounds present on auscultation. Fetal cardiotocography was within normal limits.

**Figure 1:**
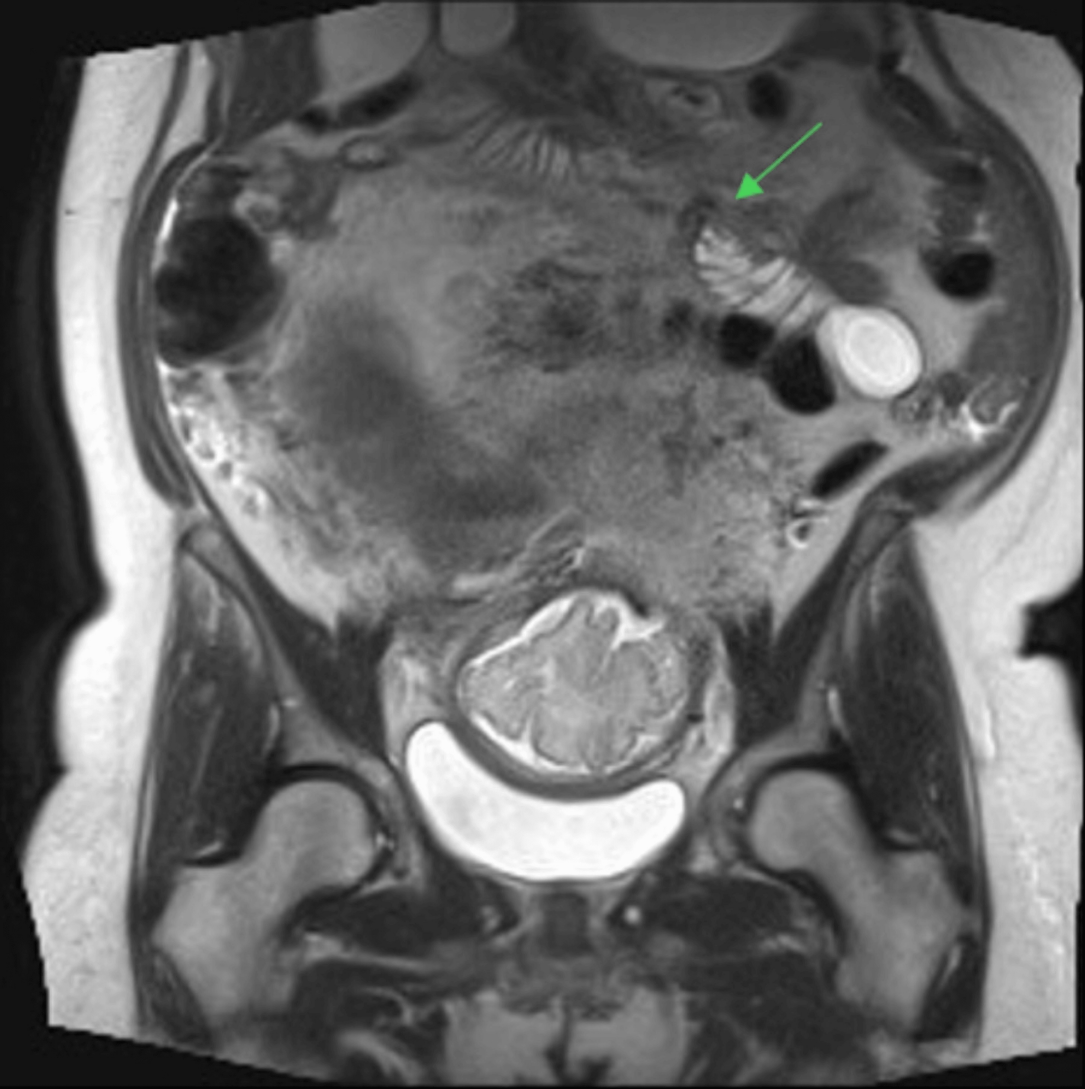
MRI T2 coronal plane demonstrating transition point from dilated to non-dilated proximal bowel loops approximately 20 cm distal to the duodenojejunal flexure

**Figure 2:**
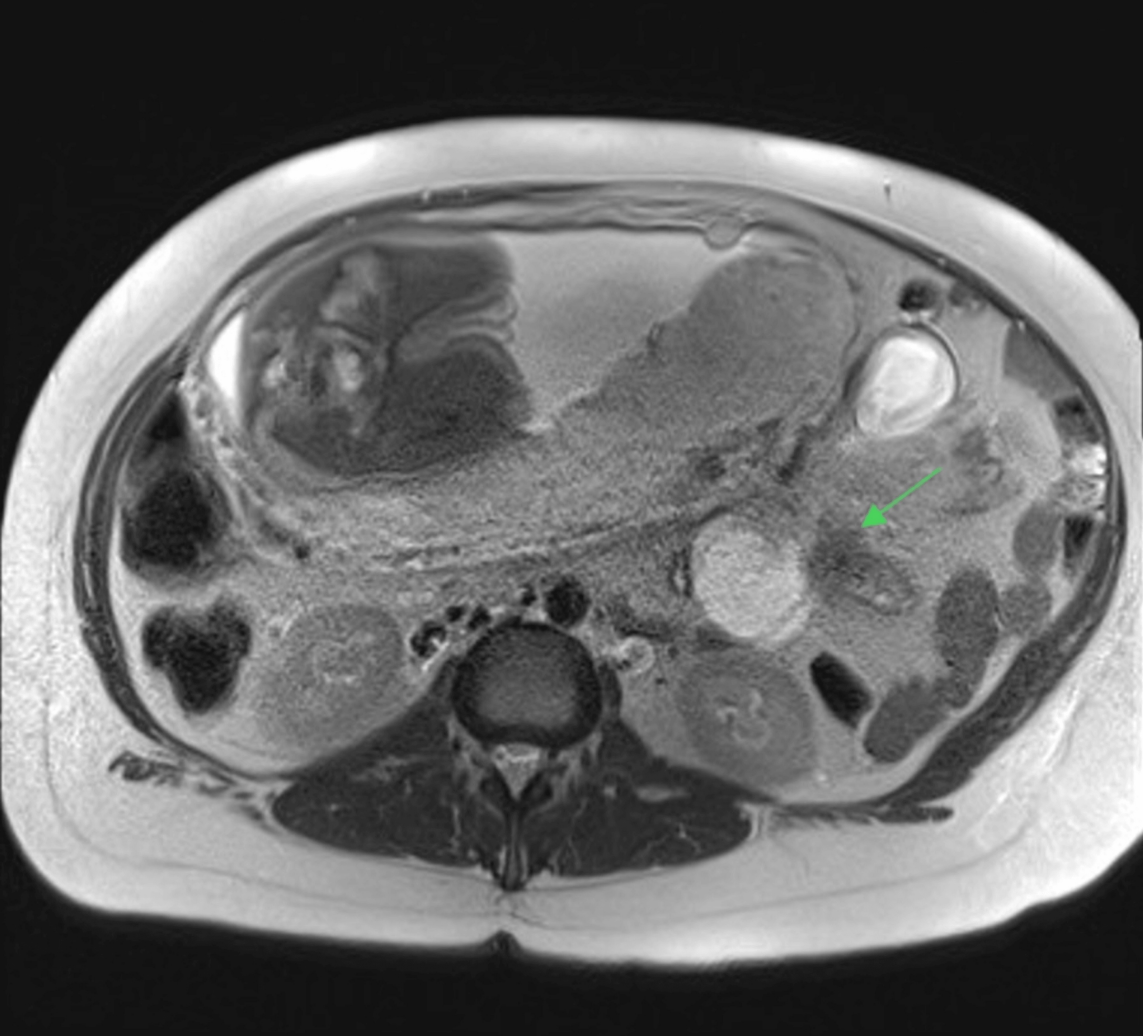
MRI T2 axial plane demonstrating internal herniation of small bowel through Petersen's space

Her bloods were unremarkable, including normal electrolytes, and she had previously had a normal abdominal ultrasound. Given a new clinical suspicion of a bowel obstruction, the patient was immediately transferred to a tertiary level centre for an MRI abdomen/pelvis with multidisciplinary input from both obstetric and surgical teams. The MRI abdomen/pelvis demonstrated a proximal bowel obstruction, with significant dilatation of the stomach, duodenum, and proximal jejunum. There was an abrupt transition to non-dilated proximal bowel loops 20 cm distal to the duodenojejunal flexure, with no mass evident (Figures 1, 2). Mild bowel wall thickening at the dilated jejunal loop raised suspicion for early ischemia; however, no intra-abdominal free gas, pneumatosis, or portal venous gas was visualized.

In the context of MRI findings consistent with a small bowel obstruction and potential ischemia, urgent surgical intervention was recommended. The patient was planned for an emergency exploratory laparotomy and possible small bowel resection. Given the risks to the fetus in the settings of possible bowel ischemia/necrosis, the decision was made to proceed with a Caesarean section.

A vertical midline laparotomy incision was made, with the fetus delivered at 32 weeks and five days gestation via caesarean section with antenatal corticosteroids administered prior. An exploratory laparotomy was subsequently performed, revealing an internal hernia through Peterson’s space with the majority of the small bowel contained within. All bowels were viable, and Peterson’s space was closed with non-absorbable sutures. The baby was admitted to the special care nursery, and the mother was discharged on day 6 post-operatively following gradual upgrading of diet.

## Discussion

Bariatric surgery is increasingly often performed in women of reproductive age, with the Roux-en-Y gastric bypass being one of the most common procedures performed. Significant and sustained weight loss is the primary advantage of bariatric surgery, which has multiple beneficial effects in pregnancy. This includes reduced risk of gestational diabetes, pregnancy-induced hypertensive disorders, preterm birth, and fetal macrosomia [9]. The disadvantages of bariatric surgery prior to pregnancy include maternal micronutrient deficiencies and increased risk of small for gestational age neonates [10]. However, one of the rarer but deleterious complications in pregnancy post-bariatric surgery is internal herniation, which can cause intestinal obstruction, bowel ischemia/necrosis, and sepsis. We presented a rare case of a small bowel obstruction secondary to a Petersen’s hernia in a pregnant patient in her third trimester with a previous Roux-en-Y gastric bypass.

Pregnant patients with internal herniation often present with post-prandial abdominal pain, nausea, and vomiting, which are relatively prevalent, non-specific symptoms in pregnancy and thus pose a diagnostic dilemma for clinicians [11]. Initial evaluation, therefore, must include a detailed history, physical examination, laboratory work, and relevant imaging modalities. In cases of internal herniation, abnormal laboratory results are rare, and ultrasound often has low sensitivity and specificity [11]. Computed tomography (CT) imaging in pregnancy is often a challenge due to concerns regarding radiation exposure, and while magnetic resonance imaging (MRI) is preferred, literature suggests that a benefit-risk analysis should be performed, as CT imaging has high specificity and sensitivity for internal herniation [3,12]. Nonetheless, internal hernias may not be detected via imaging, and thus, surgical exploration should be considered in the appropriate clinical setting.

While there are a number of mesenteric defects that can predispose pregnant patients to internal herniation post-bariatric surgery, Petersen’s space is described as the most common location for internal hernias [4]. Albeit rare, the incidence of maternal death is 9%, and fetal death is 13.6%, thus highlighting the urgent need to identify internal hernias and initiate treatment without delay [3]. Surgical exploration should be performed emergently as delays can result in lethal outcomes. If there is clinical suspicion of internal herniation, a diagnostic procedure with or without Caesarean section is recommended based on the clinical situation and risk to the fetus [8]. Intraoperatively, a thorough evaluation of bowels is needed to identify bowel necrosis and the site of herniation to rule out other competing pathologies [8]. Moreover, the current literature recommends that mesenteric defects should then be closed with non-absorbable sutures [3,8].

Significant weight reduction after bariatric surgery predisposes patients to developing internal hernias, and thus, women of reproductive age should be counselled to delay pregnancy till 12-18 months post-bariatric surgery to mitigate risk [3,11]. In the context of performing a Roux-en-Y gastric bypass, either an antecolic or retrocolic surgical approach is adopted. Current literature suggests that the major disadvantage of the retrocolic approach is that it creates more potential internal hernia sites than the antecolic approach [8]. This can predispose patients to incarcerated and strangulated hernias and downstream clinical sequelae. For example, one study demonstrated a greater incidence of internal herniation and internal obstruction in patients undergoing a retrocolic Roux-en-Y relative to an antecolic Roux-en-Y; however, this has not been investigated in pregnant patients [13]. 

## Conclusions

This case outlines a rare case of a Petersen’s hernia diagnosed in a patient with a previous Roux-en-Y gastric bypass procedure during her third trimester of pregnancy. Internal herniation must always be considered a differential diagnosis in pregnant women who present with abdominal pain and a history of previous bariatric surgery. Patient work-up and management demands a multi-disciplinary approach with involvement from both surgical and obstetric teams. Given potential deleterious maternal and fetal effects, patients often require prompt surgical intervention, with potential delivery of the fetus indicated based on the clinical situation. Albeit rare, it is paramount that there is increased awareness about the development of internal hernias in pregnancy post-bariatric surgery amongst patients and clinicians to reduce maternal and perinatal morbidity and mortality.
